# Celecoxib exhibits therapeutic potential in experimental model of hyperlipidaemia

**DOI:** 10.1371/journal.pone.0247735

**Published:** 2021-08-17

**Authors:** Martins Ekor, Phyllis Elsie Owusu Agyei, Ernest Obese, Robert Peter Biney, Isaac Tabiri Henneh, Meshack Antwi-Adjei, Ewura Seidu Yahaya, Gordon Amoakohene, Patrick Kafui Akakpo

**Affiliations:** 1 Department of Pharmacology, School of Medical Sciences, University of Cape Coast, Cape Coast, Ghana; 2 School of Pharmacy and Pharmaceutical Sciences, University of Cape Coast, Cape Coast, Ghana; 3 Department of Biomedical Sciences, School of Allied Health Sciences, University of Cape Coast, Cape Coast, Ghana; 4 Department of Pathology, School of Medical Sciences, University of Cape Coast, Cape Coast, Ghana; Max Delbruck Centrum fur Molekulare Medizin Berlin Buch, GERMANY

## Abstract

Hyperlipidaemia is a major risk factor for cardiovascular diseases, the leading cause of death globally. Celecoxib attenuated hypercholesterolaemia associated with CCl_4_-induced hepatic injury in rats without improving liver function in our previous study. This present study investigated the lipid lowering potential of celecoxib in normal rats fed with coconut oil subjected to five deep-frying episodes. Male Sprague Dawley rats were randomly assigned to groups (n = 6 rats/group) which received physiological saline (10 mL/kg), unheated coconut oil (UO, 10 mL/kg) or heated coconut oil (HO, 10 ml/kg) for 60 days. Groups that received HO were subsequently treated with either physiological saline, atorvastatin (25 mg/kg), celecoxib (5 mg/kg) or celecoxib (10 mg/kg) in the last fifteen days of the experiment. Rats were sacrificed 24 hours after last treatment and blood and tissue samples collected for analysis. HO consumption produced significant hyperlipidaemia and elevation in marker enzymes of hepatic function. Celecoxib ameliorated the hyperlipidaemia as shown by the significantly (P<0.05) lower total cholesterol, triglycerides, low and very low density lipoprotein in the celecoxib-treated rats when compared with HO-fed rats that received saline. Celecoxib also reduced (P<0.05) alanine aminotransferase, aspartate aminotransferase, alkaline phosphatase and liver weight of hyperlipidaemic rats. Similarly, hepatocellular damage with the hyperlipidaemia was significantly reversed by celecoxib. However, serum TNF-α and IL-6 did not change significantly between the various groups. Taken together, data from this study suggest that celecoxib may exert therapeutic benefit in hyperlipidaemia and its attendant consequences.

## Introduction

Hyperlipidaemia, also recognised as dyslipidaemia, describes the manifestation of different disorders of lipoprotein metabolism [1]. Patients with hyperlipidaemia are mostly asymptomatic but have an increased risk for cardiovascular diseases (CVDs). CVDs are recognized as one of the leading causes of mortality and morbidity worldwide [2–6]. Atherosclerosis, a vascular disease affecting blood circulation in the coronary, central, and peripheral arteries, is the major form of CVD and it is characterized by chronic inflammatory build up, driven largely by lipid accumulation within the walls of the artery. Unlike acute inflammation, atherosclerosis is hallmarked by a state of unresolved low-grade chronic inflammation [3]. Besides hypertension, chronic dyslipidaemia is a major cause of atherosclerosis [7,8].

Hyperlipidaemia may affect the severity of tissue damage in other pathological conditions, notably in liver injury [9]. Primary associated clinical findings of fatty liver are hyperlipidaemia, hyperglycaemia, hypertension, and hyperuricaemia [10]. Although, some success has been achieved with the use of statins in the management of hyperlipidaemia [11], the use of statins has been associated with side effects such as myopathy, headache, bowel upset, nausea, sleep disturbance, increased creatinine phosphokinase and serum transaminase hence requiring routine monitoring of these parameters [12]. Fibrates, bile acid sequestrants and nicotinic acid which constitute the major alternative modalities of treatment also have some side effects [12]. This notwithstanding, their control of lipid levels is far from satisfactory and thus calls for increased search for newer drugs with hypolipidaemic properties or repurposing of existing drugs for use in hyperlipidaemic conditions.

The non-steroidal anti-inflammatory drugs (NSAIDs) are used primarily for the management of inflammatory conditions such as arthritis and are known to exert their effect via inhibition of cyclooxygenase (COX-1 and COX-2) activity [13]. At higher concentrations, NSAIDs also reduce production of superoxide radicals, induce apoptosis, reduce adhesion molecules expression, decrease nitric oxide synthase, decrease pro-inflammatory cytokines (*e*.*g*., TNF-α, interleukin-1) and alter cellular membrane functions [14]. All these markers are known to be up-regulated in inflammatory conditions and other disorders which have inflammatory subsidiaries. Some NSAIDs such as ibuprofen have been shown to lower plasma cholesterol levels and reduce the progression of atherosclerosis [15–17] while others like indomethacin lower cholesterol content in liver and atherosclerotic blood vessels [18,19].

The coxibs, designed to selectively block COX-2, appeared a promising solution in the effort to avoid the gastrointestinal and other adverse effects that were noted with traditional NSAIDs [20,21]. Celecoxib was the first specific COX-2 inhibitor approved for the treatment of rheumatic diseases. Observations from several clinical studies have led to concerns about the cardiovascular safety of the COX-2 inhibitors. While the evidence regarding the cardiovascular risk associated with these drugs was not encouraging, a number of studies demonstrated that celecoxib is safer than other coxibs and indeed several studies have shown that celecoxib is capable of exerting a beneficial impact on cardiovascular health [22–25].

There has been a general assumption that COX-2 inhibitors may be beneficial in atherosclerosis, liver disease and hypercholesterolaemia since pathogenesis of these diseases is closely linked with prostaglandins [15] and since upregulation of COX-2 expression has also been demonstrated in hyperlipidaemia [26]. In our previous study, celecoxib was observed to significantly attenuate hypercholesterolemia and lipid peroxidation associated with liver injury during carbon-tetrachloride–associated hepatotoxicity in rats [27]. This important observation needs further evaluation in experimental hyperlipidaemia models devoid of hepatotoxin to ascertain the possible therapeutic potential of celecoxib in hyperlipidaemia. Considering the huge cost, time, safety and legal challenges associated with discovery of newer drugs, repurposing of already existing drugs in clinical use for newer indications provides rapid alternative to ensure improved access to medicines with relatively minimal resources. It is on this basis that we evaluate the FDA-approved selective COX-2 inhibitor, celecoxib, as a potential addition to the already existing pharmacotherapy for hyperlipidaemia in the current study.

## Materials and methods

### Animals

Male Sprague-Dawley rats (170–250 g) were obtained from the Noguchi Memorial Institute for Medical Research, Ghana. The animals were housed in stainless cages (34 × 47 × 18) in groups of five at the animal house facility of School of Biological Sciences, University of Cape Coast. Animals were fed with normal commercial diet bought from Flour Mills of Ghana Limited, Tema, Ghana and water was provided *ad libitum*. They were kept under normal laboratory conditions with regards to room temperature and humidity. All the techniques and protocols used in the study were done in accordance with established public health guidelines in “Guide for Care and Use of Laboratory Animals” [28] and approved by the Research and Ethics Committee of the School of Pharmacy and Pharmaceutical Sciences, University of Cape Coast (approval number UCCSoPPS/REC/18/004).

### Drugs and chemicals

Celecoxib (Celebrex^™^) and Atorvastatin (Lipitor^®^) were purchased from Pfizer Pharmaceutical LLC, Vega Baja, Puerto Rico Virgin^®^ coconut oil was purchased from the Kotokuraba market at Cape Coast, Ghana.

### Experimental design and treatment

Thirty-six rats (weighing between 170–250 g) were divided into six groups of 6 rats per group and fed with normal commercial diet during the 7-day acclimatization period and throughout the 60-day experimental period. Animals were treated as follows as shown in the Table 1. The required doses of the oil, normal saline (NS, 10 mL/kg), celecoxib (CXB, 5 and 10 mL/kg) and atorvastatin (ATO, 25 mL/kg) were administered orally by the use of an oral gavage. Celecoxib and atorvastatin were administered as emulsions with tween 80 *q*. *s*. as surfactant whereas the corresponding NS as additional treatment contained 1% tween 80. The doses of CXB were selected according to previous studies [27]. Administration were done such that animals received oil/NS in the morning (06:00 GMT) and CXB or ATO or NS (containing 1% tween 80) in the evening at 18:00 GMT. This was done to ensure that animals received minimum stress at each drug administration and also to prevent possible interferences in drug absorption. The various groups were then treated as follows: Group 1: physiological saline (NS, 5 mL/kg/day) plus additional NS in 1% tween 80 (10 mL/kg/day) from 46th–60th day; Group 2: unheated oil (UO, 5 mL/kg/day) for 45 days plus additional NS in 1% tween 80 (10 mL/kg/day) from the 46th–60th day; Group 3: heated oil (HO, 5 mL/kg/day) for 45 days plus NS in 1% tween 80 (10 mL/kg/day) from the 46th– 60th day; Group 4: HO (5 mL/kg/day) for 45 days plus atorvastatin (25 mg/kg/day) from the 46th -60th day; Group 5: HO (5 mL/kg/day) for 45 days plus celecoxib (5 mg/kg/day) from the 46th–60th day and Group 6: HO (5 mL/kg/day) for 45 days plus celecoxib (10 mg/kg/day) from the 46th -60th day. Animals were fasted overnight after all the treatments and fasting blood glucose levels were measured 24 h after the final treatment on the 60^th^ day. The animals were humanely sacrificed by cervical dislocation and blood and organs were harvested for other investigations. Blood samples were collected via cardiac puncture into EDTA and gel separator tubes for haematological and biochemical analyses, respectively.

**Table 1:** Treatment schedule.

	Group 1	Group 2	Group 3	Group 4	Group 5	Group 6
**Treatment for 60 Days**	NS 5 mL/kg	UO 5 mL/kg	HO 5 mL/kg	HO 5 mL/kg	HO 5 mL/kg	HO 5 mL/kg
**Additional Treatment from 46** ^**th**^ **to 60** ^**th**^ **Day**	NS 10 mL/kg	NS 10 mL/kg	NS 10 mL/kg	ATO 25 mg/kg	CXB 5 mg/kg	CXB 10 mg/kg

NS = normal saline, UO = unheated oil, HO = heated oil, ATO = atorvastatin and CXB = celecoxib.

Group 1 (naïve control); Group 2 (unheated oil control); Group 3 (heated oil/negative control); Group 4 (positive control); Group 5 (low dose celecoxib) and Group 6 (high dose celecoxib).

#### Total body weight relative weight of organs

The total body weight of animals were recorded every ten (10) days. Additionally, on the 61^st^ day, specific organs including the liver, heart, kidney, lungs and spleen were harvested and weighed. Relative organ weights (mg/kg body weight) were estimated and values analyzed.

#### Haematological analysis

Blood samples were analyzed by haem automated analyzer (CELL-DYN 1700, Abbot Diagnostics Division, Abbot Laboratories, Abbot Park, Illinois, USA) for total blood count and specific differentials.

#### Biochemical analysis

Blood samples were allowed to clot for 30 min at room temperature and centrifuged at 1000 rpm for 10 min. Serum obtained was stored at -20°C until biochemical analysis was carried out. Serum indices were analyzed by an automated analyzer (ATAC 8000 Random Access Chemistry System, Elan Diagnostics, Smithfied, RI, USA) and estimations for aspartate aminotransferase (AST), alanine aminotransferase (ALT), alkaline phosphatase (ALP), Creatinine, blood urea nitrogen (BUN), fasting blood sugar (FBS), total cholesterol (TC), triglycerides (TG), high density lipoprotein (HDL) cholesterol direct, low-density lipoprotein (LDL) cholesterol and very low-density lipoprotein (VLDL) were recorded.

#### Serum cytokine (IL-6 and TNF-α) levels

The blood samples were centrifuged at 1000 rpm for 10 min. Sera formed were aliquoted into eppendorf tubes and stored at -20°C before analysis. Serum levels of IL-6 and TNF-α were estimated in duplicates with specific rat ELISA kit (Boster Biological Technology 3942 Valley Ave Pleasanton, CA 94566, USA) assay in accordance with the recommendations of the manufacturer. The absorbance of the samples was read at 450 nm using a micro-plate spectrometer (Spectramax 190 Micro-plate Spectrometer, 90-250V 50-60Hz, Molecular Devices, CA, USA).

#### Histopathological studies

Portions of the tissues from liver were used for histopathological examination. Tissues were fixed in 10% neutral buffered formalin (pH 7.2) and dehydrated through a series of ethanol solutions, embedded in paraffin and routinely processed for histological analysis. A section (2 μm thickness) was cut and stained with haematoxylin-eosin for examination. The stained tissues were observed through an Olympus BX-51 microscope (Olympus Corporation, Tokyo, Japan) and photographed by Infinity 4 USB Scientific Camera (Lumenera Corporation, Otawa, Canada).

### Data analysis

Data has been presented as mean of six rats ± standard error of mean (SEM). The presence of significant differences between means of groups was determined by one-way analysis of variance (ANOVA) using GraphPad Prism for Windows version 7 (GraphPad Software, San Diego, CA, USA). Significant difference between groups was determined using the Newman-Keuls’ Multiple Comparison Test with *P* < 0.05 considered statically significant. Time-course curves were analysed using two way ANOVA followed by Tukey’s post *hoc* test.

## Results

### Changes in total body and relative organ weights

**Fig 1:**
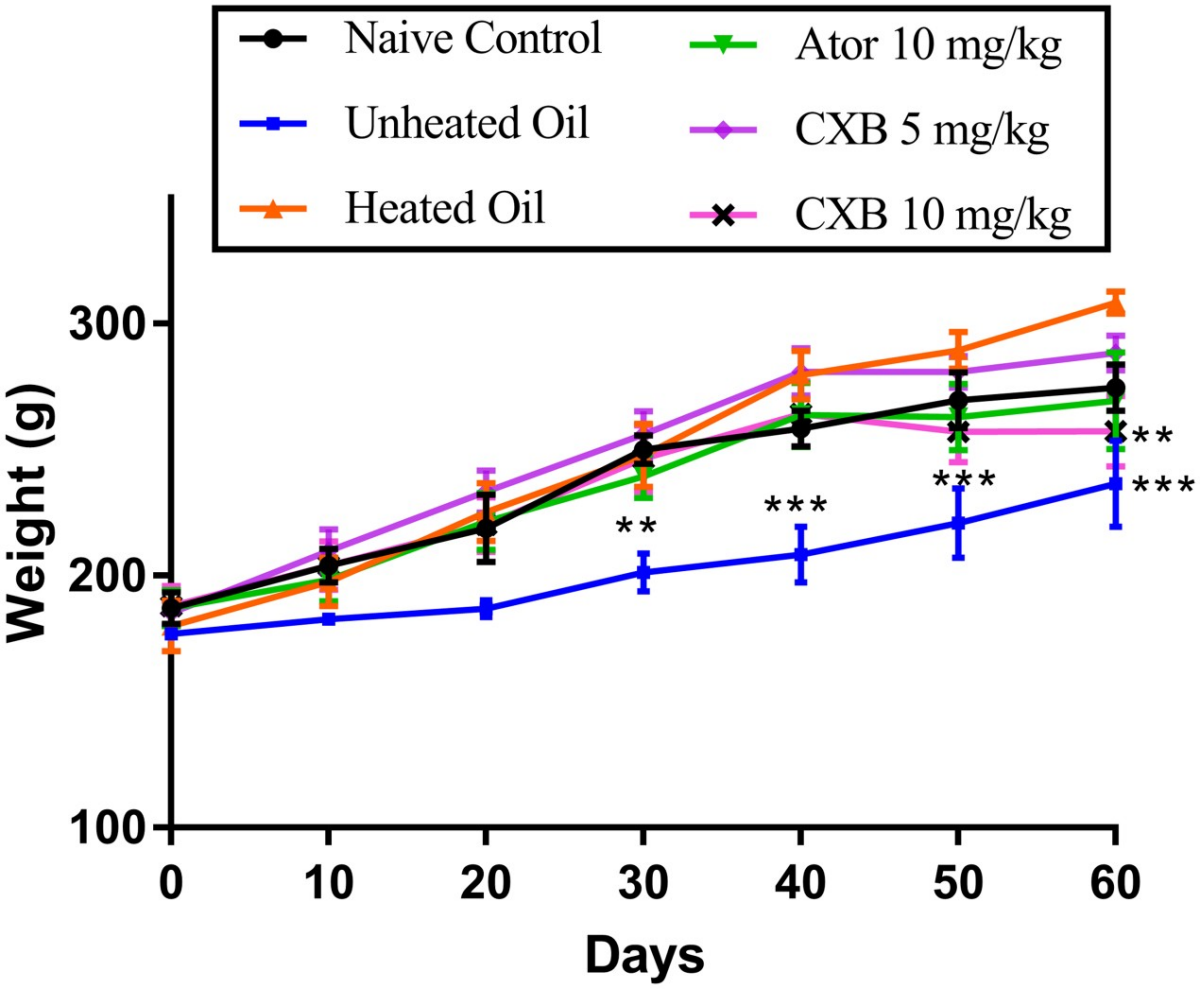
Effect of celecoxib (CXB 5 and 10 mg/kg) and atorvastatin (ATO 25 mg/kg) on the total body weight of rats. The symbol ** and *** represent significant differences (*P <* 0.01 or *P <* 0.001 respectively) between treatment groups and heated oil only group (all were compared using two-way ANOVA followed by Tukey’s post *hoc* test).

The results presented in Fig 1 describe the effect of celecoxib (CXB 5 and 10 mg/kg) and atorvastatin (ATO 25 mg/kg) on the total body of rats previously administered with heated coconut oil. Administration of unheated oil to the rats significantly caused a decrease in total body weights of rats at day 30 (*P* < 0.01), and days 40–60 (*P* < 0.001) compared to the heated oil only treatment group. Also, a significant difference (*P* < 0.01) from the heated oil-only (negative control) group was observed only at day 60 in the rats treated with the CXB 10 mg/kg. The rest of the treatments did not cause any significant changes in the total body weights of rats in other treatment groups in comparison with the negative control (heated oil only treatment group).

**Fig 2:**
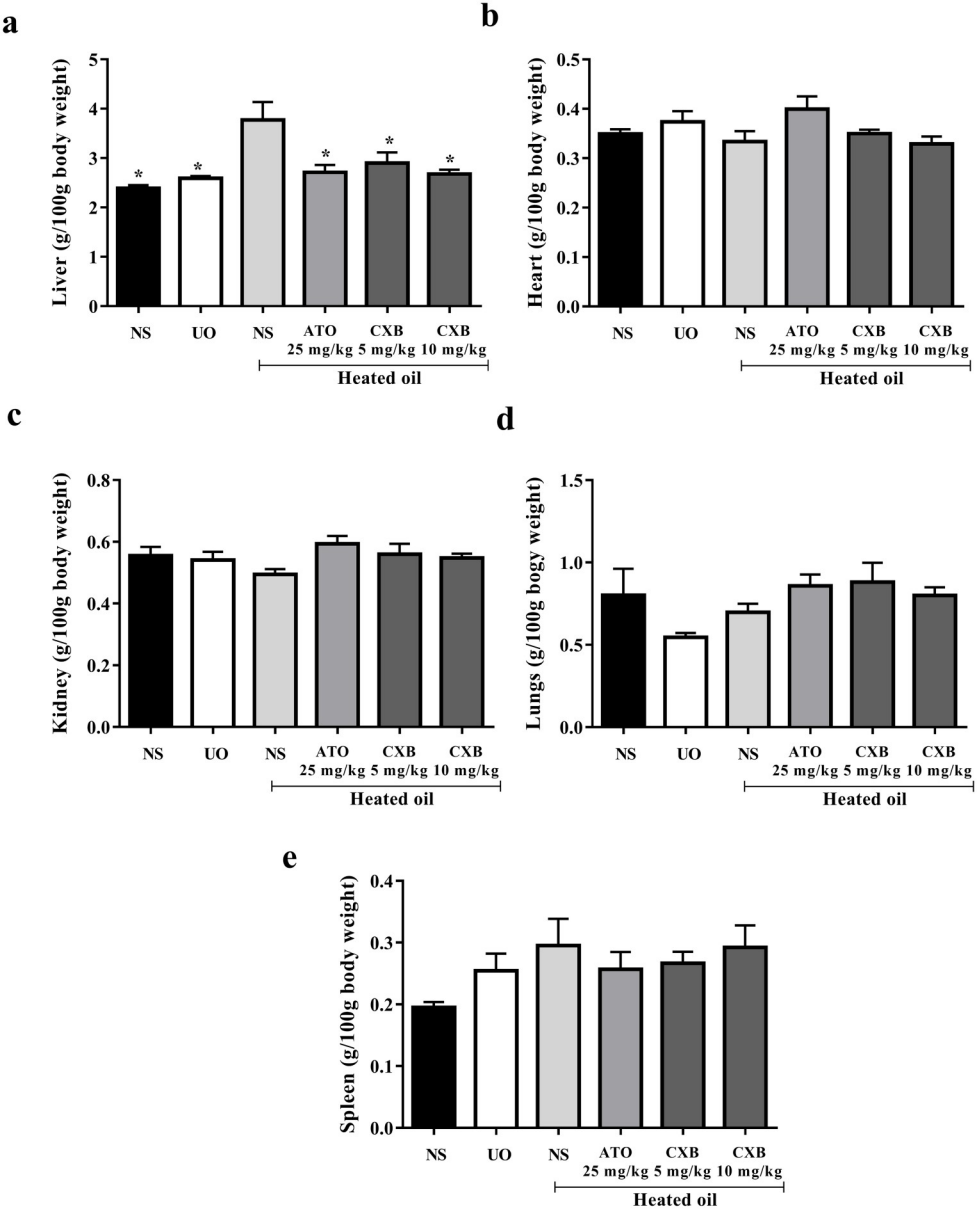
Effect of celecoxib (CXB 5 and 10 mg/kg) and atorvastatin (ATO 25 mg/kg) on the weight of (a) liver, (b) heart (c) kidney (d) lungs and (e) spleen in heated-oil induced hyperlipidaemia model in Sprague-Dawley rats. Values are expressed as mean ± SEM (n = 6). The symbol * represents significant differences between treatment groups and heated oil only group (*P <* 0.05) (all were compared using one-way ANOVA followed by Newman-Keuls’ post *hoc* test).

The results presented in Fig 2 also show that the relative liver weights (liver-to-body weight ratio) of the group that received only heated oil was significantly (*P* < 0.05) higher compared to the naïve control and unheated oil group. This was, however, significantly (*P* < 0.05) decreased by treatment with celecoxib and atorvastatin. The relative weights of other organs such as heart, kidney, lungs and spleen were not significantly affected as shown in Fig 2.

### Changes in haematological parameters

**Fig 3:**
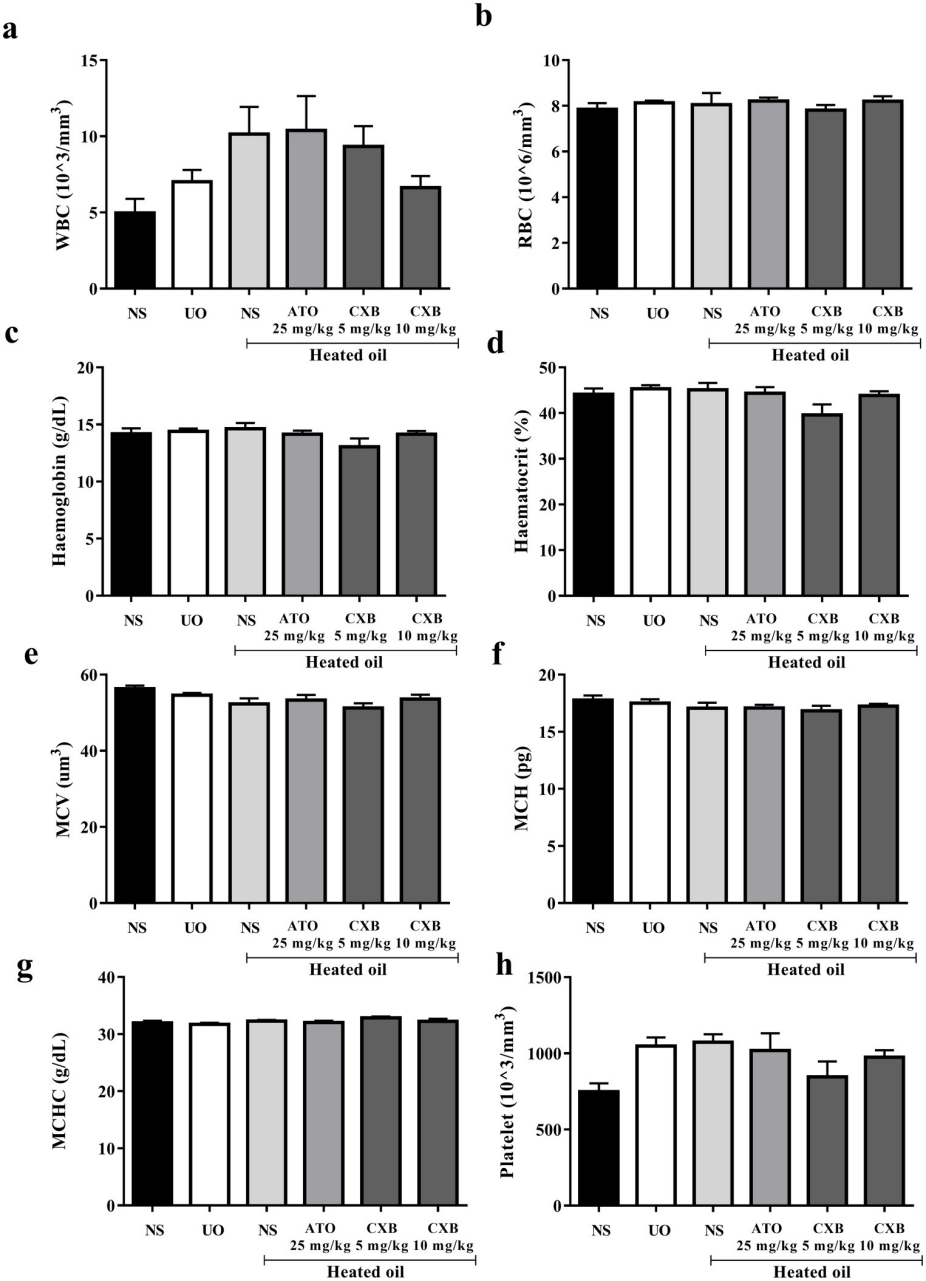
Effect of celecoxib (CXB 5 and 10 mg/kg), atorvastatin (ATO 25 mg/kg) on haematological parameters such as (a) white blood cells (b) red blood cells (RBCs) (c) haemoglobin (d) haematocrit (e) mean cell volume (MCV) (f) mean cell haemoglobin (g) mean cell haemoglobin concentration (MCHC) and (h) platelet in overheated-oil induced hyperlipidaemia in Sprague-Dawley rats. Values are expressed as mean ± SEM (n = 6). There were no significant differences between treatment groups and the various controls.

The heated oil and the various drug treatments did not significantly (*P* > 0.05) alter haematological parameters such as the red blood cell count, haemoglobin, haematocrit, mean cell volume, mean cell haemoglobin concentration, platelet count, white blood cell count Fig 3.

### Changes in serum biochemical parameters

**Fig 4:**
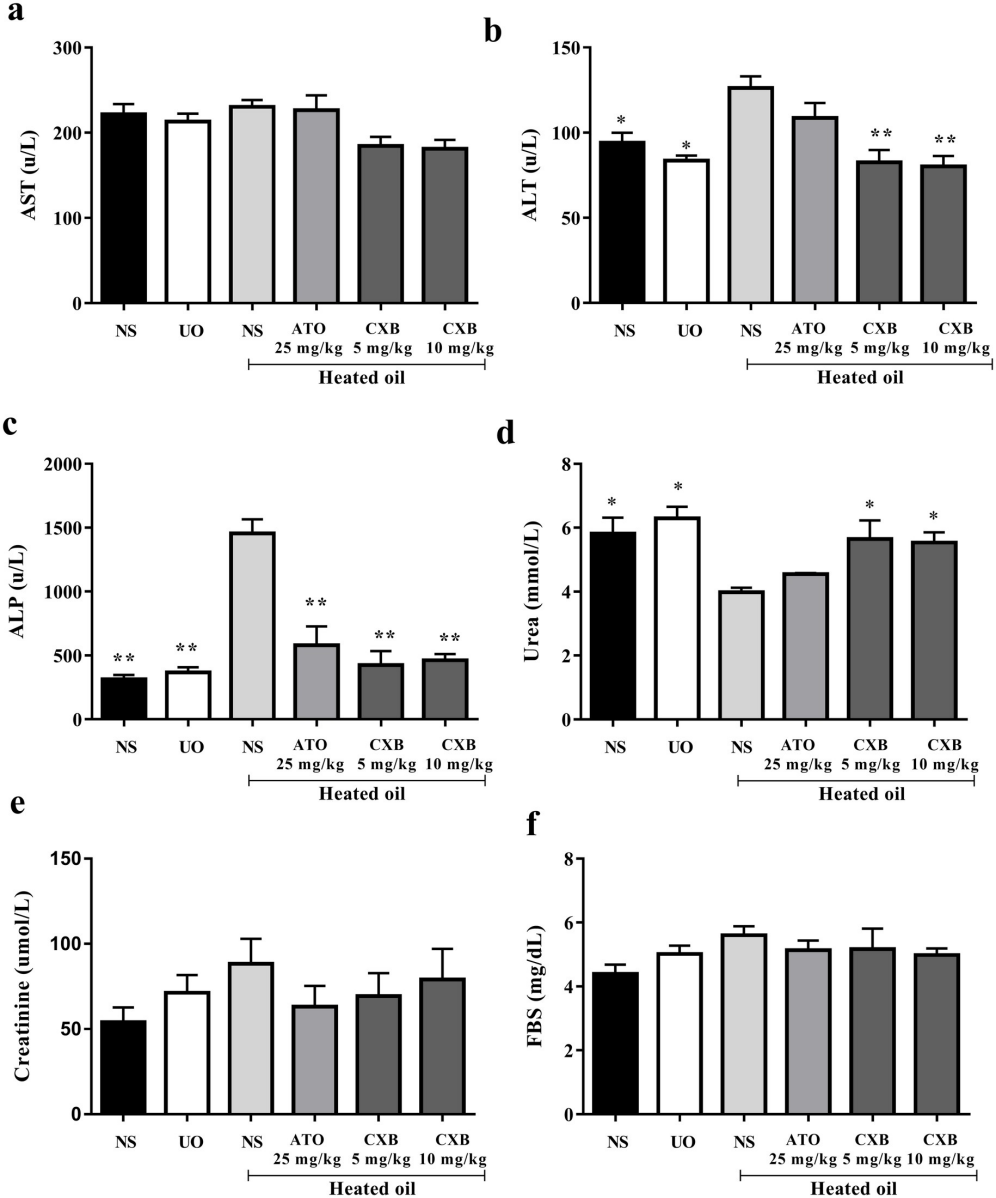
Effect of celecoxib (CXB 5 and 10 mg/kg), atorvastatin (ATO 25 mg/kg) on serum lipid parameters such as (a) AST (b) ALT (c) ALP and (d) urea; and (e) fasting blood sugar in overheated-oil induced hyperlipidaemia in Sprague-Dawley rats. Values are expressed as mean ± SEM (n = 6). The symbols * and ** represents significant differences (*P* < 0.05 and *P* < 0.01 respectively) between treatment groups and heated oil only group (all were compared using one-way ANOVA followed by Newman-Keuls’ post *hoc* test).

Results presented in Fig 4 show alanine aminotransferase (ALT) and alkaline phosphatase (ALP) activities of rats fed with heated coconut oil were significantly (*P* < 0.05) higher than those of the naïve control. However, treatment with celecoxib (5 and 10 mg/kg) significantly (*P* < 0.01) reversed these elevations in the liver enzymes. Activities of aspartate aminotransferase (AST) enzyme as well as fasting blood glucose were however, not significant different among the various treatment groups.

With respect to urea however, treatment of rats with heated oil significantly reduced its levels compared to the naïve control and those treated with unheated oil. The decrease was however, significantly (*P* < 0.05) reversed to normal by both doses of celecoxib but not atorvastatin (Fig 4).

### Changes in lipid profile

**Fig 5:**
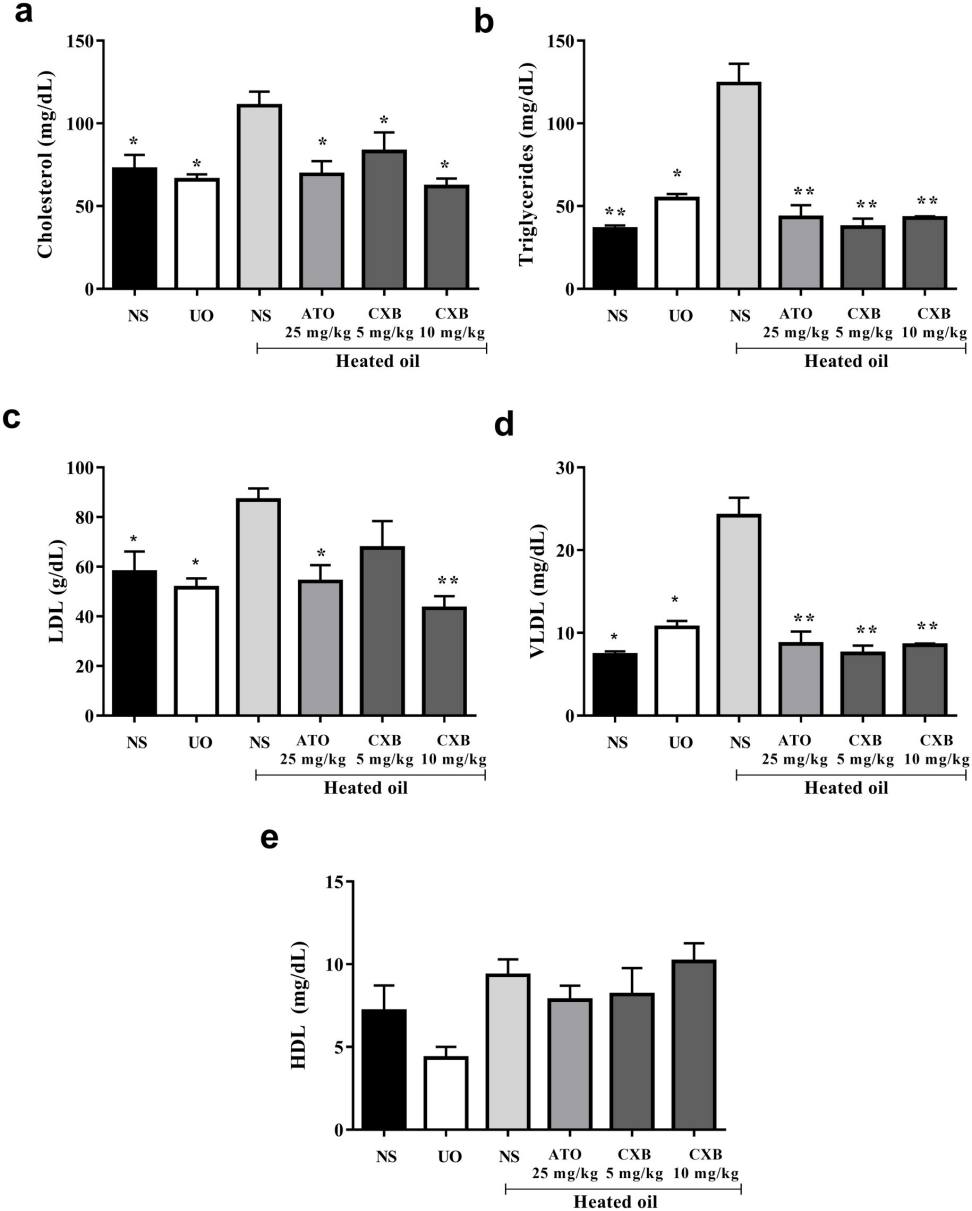
Effect of celecoxib (CXB 5 and 10 mg/kg), atorvastatin (ATO 25 mg/kg) on serum lipid parameters such as (a) cholesterol (b) triglycrides (c) low density lipoprotein (d) very low density lipoprotein and (e) high density lipoprotein cholesterol in overheated-oil induced hyperlipidaemia in Sprague-Dawley rats. Values are expressed as mean ± SEM (n = 6). The symbols * and ** represents significant differences (*P <* 0.05 and *P <* 0.01 respectively) between treatment groups and heated oil only group (all were compared using one-way ANOVA followed by Newman-Keuls’ post *hoc* test).

Treatment of rats with heated oil only significantly (*P* < 0.05) elevated the levels of cholesterol, triglycerides, LDL as well as VLDL. All these parameters were significantly reversed by treatment with atorvastatin and celecoxib. The levels of high density lipoproteins (HDL) were not significantly affected compared to the controls despite the fact that the levels decreased in rats treated with unheated oil only (Fig 5).

### Changes in cytokine levels

**Fig 6:**
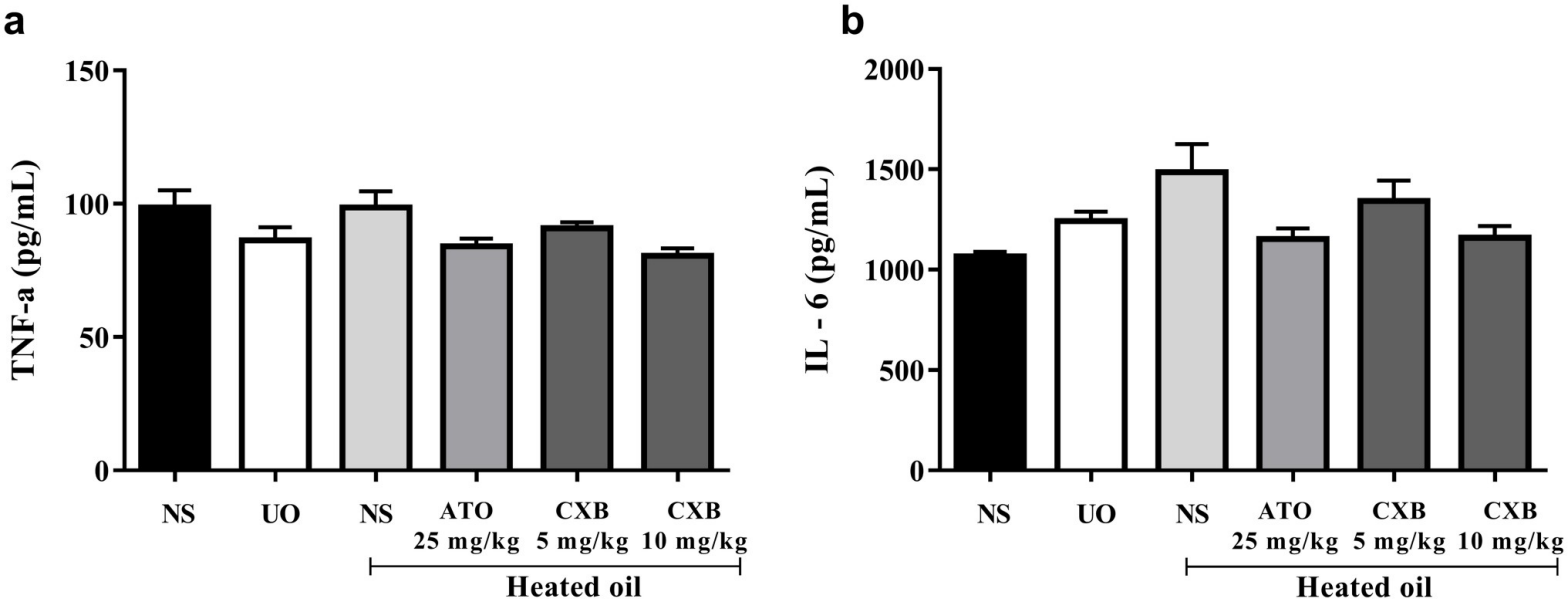
Effect of celecoxib (CXB 5 and 10 mg/kg) and atorvastatin (ATO 25 mg/kg) on the levels of serum cytokines such as (a) TNF-α and (b) IL-6 in overheated-oil induced hyperlipidaemia in Sprague-Dawley rats. Values are expressed as mean ± SEM (n = 6). There were no significant differences between heated oil and other treatment groups (all were compared using one-way ANOVA followed by Newman-Keuls’ post *hoc* test).

Results presented in Fig 6 show that treatment of rats with heated coconut oil did not induce significant changes in the levels both TNF-α as well as interleukin 1β compared with naïve control group. Additionally, the celecoxib as well as atorvastatin did not alter the levels of the cytokines significantly (Fig 6).

### Histopathological changes in the liver

**Fig 7:**
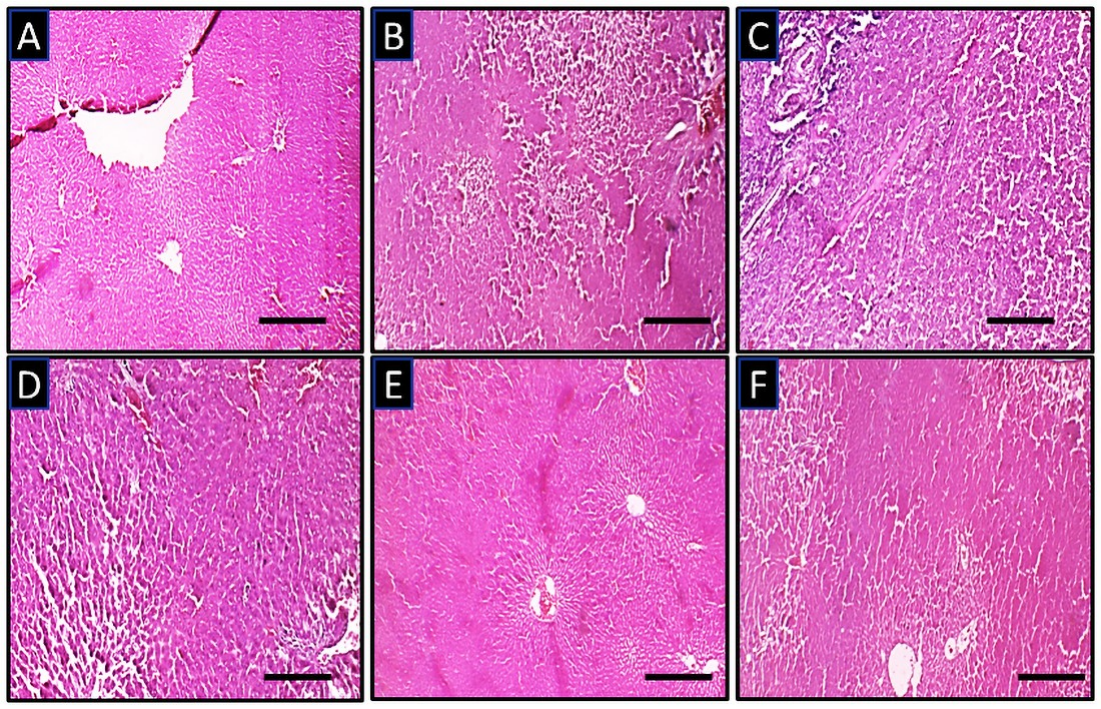
Representative photomicrographs of the liver of experimental animals showing the general histomorphology. (A) naïve control group (B) unheated oil treated group (C) heated oil only treated group as negative control, (D) heated oil in addition to atorvastatin 25 mg/kg (E) heated oil in addition to celecoxib 5 mg/kg and (F) heated oil in addition to celecoxib 10 mg/kg. Scale bar is 50μ.

Photomicrographs presented in Fig 7 show that the naïve control (A) and unheated oil group (B) present with typical histomorphology characterized by densely packed hepatocytes with normal staining intensity, cellularity, with no apparent pathological alterations. Group C (heated oil only treated group) and group D (heated oil plus atorvastatin 25 mg/kg treated group) present with darkly stained nuclei suggesting pyknotic changes. Groups E and F representing animals treated with heated oil as well as celecoxib (5 mg/kg and 10 mg/kg respectively) also show characteristically normal hepatic histomorphology.

## Discussion

The global increase in the incidence of cardiovascular events continues to present a major public health issue because treatment remains suboptimal. Evidence abounds that lipid lowering therapy with statins (or ezetimibe in combination with a statin) contributes to reducing major adverse cardiovascular events. In spite of this, substantial risk of cardiovascular events remains even among patients receiving statin therapy whose LDL-C is <70 mg/dL [29,30]. Alternative strategies are also required to lower lipids in patients who experience adverse effects on maximally tolerated statin therapy. These challenges call for innovations in the field of dyslipidaemia to address the several areas of unmet need [29].

Recent studies have demonstrated the association between increase in the expression of COX-2 and the development of metabolic disorders including obesity, diabetes mellitus, and non-alcoholic fatty liver disease (NAFLD). Studies have also shown that COX-2 activity not only has influence on insulin sensitivity [31], but also acts as pro-inflammatory mediator during the progression of NAFLD [32]. The latter has gained prominence as part of the possible mechanisms contributing to the protective effect of celecoxib against the development of NAFLD. Many studies suggested that celecoxib could attenuate liver steatosis and inflammation in NAFLD [33]. We also observed the ability of celecoxib to lower plasma cholesterol and attenuate hepatic lipid peroxidation in CCl_4_-mediated hepatotoxicity in rats [27]. In this study, we examined if the observed hypocholesterolaemic property of celecoxib in our previous study was unrelated to its hepatoprotective effect by evaluating its lipid lowering potential in rats fed with high fat (heated coconut oil) and without chemically-mediated hepatic injury.

The results presented in Fig 2 describe the effect of celecoxib (CXB 5 and 10 mg/kg) and atorvastatin (ATO 25 mg/kg) on hyperlipidaemia induced by heated coconut oil. Naïve control group received only normal saline (10 mL/kg) throughout the experiment, the negative control group received heated oil and normal saline (10 mL/kg) whereas another group received unheated oil and normal saline (10 mL/kg). The relative liver weight (liver-to-body weight ratio) of the group that received only heated oil was significantly (*P* < 0.05) higher compared to the naïve control and unheated oil group. This was, however, significantly (*P* < 0.05) decreased by treatment with celecoxib and atorvastatin. The relative weights of other organs such as heart, kidney, lungs and spleen were not significantly affected as shown in Fig 2. Generally, when oil is subjected to high temperature heating, free radicals are generated [34]. This may lead to several pathological changes in some organs as seen in the significantly increased weight of the liver. This remarkable increase in liver-to-body weight ratio has been attributed to the ability of the oil to increase liver microsomal lipid composition resulting in fatty liver [35]. Importantly, both celecoxib and atorvastatin significantly reversed the liver weight to normal which suggests a possible reduction in the fat accumulation in the liver.

Earlier reports suggest that there is a strong correlations (both positive and negative) between the haematological parameters and the different lipid parameters [36,37]. Despite this fact, none of the haematological parameters assessed in the study was significantly affected (Fig 2).

Results presented in Fig 3 show alanine aminotransferase (ALT) and alkaline phosphatase (ALP) activities of rats fed with heated coconut oil were significantly (P<0.05) higher than those of the naïve control. This is an indication of a possible hepatocellular damage [38]. Despite both atorvastatin and celecoxib were able to reverse the anomaly in the levels of ALP, only celecoxib was able to significantly reverse the ALT levels to normal. The ALT enzyme is distributed in many tissues, but higher levels are present in the liver with elevated serum levels found in hepatocellular disorders than in intrahepatic or extra-hepatic cholestatic disorders. Celecoxib (5 and 10 mg/kg) significantly (*P* < 0.01) reversed this effect and this could be pointing to an ameliorative or protective effect of celecoxib against liver dysfunction associated with hyperlipidaemia. This results also confirm the possible involvement of liver disease and hypercholesterolaemia [27,38].

Furthermore, we observed that sub-chronic administration of heated oil also produced a significant decrease in blood urea levels in the rats in the negative control compared to the naïve control. Though reduced protein intake and pregnancy could account for physiological decrease in serum urea levels [39], these factors were ruled out as all animals used in our study were given same protein-rich diet and none of them was pregnant. However, ample evidence suggests that pathological cause of reduced serum urea concentration is largely confined to severe liver disease [39,40]. This reflects the central role that the liver plays in urea production via the urea cycle. As such, a compromised liver could result in a decreased production of urea and a consequent reduction in serum urea concentration. This decrease was significantly reversed by celecoxib, but not atorvastatin, which suggests the former played a role in protecting the liver thereby restoring urea production capacity of the liver to normal. Already, the hepatoprotective effect of celecoxib has been reported by our group (27). However, this hepatoprotective was as not observed in the group that was treated with atorvastatin. This also suggests that, unlike celecoxib, the hypolipideamic effect of atorvastatin is devoid of a significant hepatoprotective activity.

Rather obvious and significant (*P* < 0.05) was the increase in total cholesterol, triglyceride, LDL, and VLDL levels in the heated oil treated group as shown in Fig 5. Treatment with atorvastatin and celecoxib significantly (*P* < 0.05) ameliorated this effect. Hyperlipidaemia with a noticeable increase in low-density lipoprotein (LDL) cholesterol levels is common in patients with chronic cholestatic liver disease [40]. Therefore, the corresponding increase in ALT is not surprising though an increase in AST should have been expected. Some lipoproteins (notably those containing apoprotein B-100) are retained in the sub-endothelial space, by means of a charge-mediated interaction with extracellular matrix and proteoglycans [41]. This allows reactive oxygen species to modify the surface phospholipids and unesterified cholesterol of the small LDL particles. Because of LDL oxidation, isoprostanes are formed [42]. Vasoconstriction in the setting of high levels of oxidized LDL appear to be associated with a reduced release of the vasodilator nitric oxide from the damaged endothelial wall as well as increased platelet aggregation and thromboxane release [38,43]. The state of hypercholesterolaemia leads invariably to an excess accumulation of oxidized LDL within the macrophages, thereby transforming them into "foam" cells. The rupture of these cells can lead to further damage of the vessel wall due to the release of oxygen free radicals, oxidized LDL, and intracellular enzymes [43].

Abnormal production of some cytokines such as tumour necrosis factor (TNF)-α, interleukin-1-beta (IL- 1β), soluble IL-2 receptor (sIL-2R), IL-6, and chemokine IL-8 have been implicated in the pathogenesis of various inflammatory and autoimmune diseases [43]. When the sera of animals treated with heated oil were tested for serum cytokines (IL-6 and TNF-α), levels were not significantly (*P* > 0.05) affected as shown in Fig 6. Though many *in vivo* studies have demonstrated that TNF-α and IL-6 are important components of the pro-inflammatory response [44], this was not observed in our study.

Since the liver was the only organ whose relative weight was significantly affected by the various treatments, it was expedient to conduct a histopathological study on them. This falls in line with the recommendation of the Society for Pathology and Toxicology (STP) that organ weights should be interpreted alongside hisptopathology [45]. Results obtained from the histology of the tissues from animals that received heated oil only as presented in Fig 7 showed a distorted architecture of the liver with pyknotic nuclei. These changes were not observed in the naïve control groups as well as the group that received unheated oil. Treatment with 5 mg/kg and 10 mg/kg celecoxib reduced this damage to a very large extent. However, the histology of the group that received atorvastatin was not significantly different from the heated oil only group. Though the histological differences between the heated oil only group and the celecoxib groups were quite moderate, there were significant differences in liver enzymes such as ALT and ALP which are important markers of liver damage. It is worth noting that despite elevated transaminase activities is considered as an important marker of liver damage, a drastic elevation of serum activities of liver enzymes markers ought not necessarily to reflect liver cells death with its associated marked alterations in histomorphology [46].

The present study has some few limitations and we believe that these challenges will create perspectives for future direction. First, we were constrained to using six animals per group for our study due to ethical considerations. The strength of data interpretation and statistical significance may be influenced by this sample size, although reliance on statistical significance in biological experiments remains controversial. Secondly, the animals received the various treatments orally via the oral gavage method. This was done to ensure that all animals receive the required quantities of the oil and drugs. Although we do acknowledge that this may have induced some stress in the animals, we ensured that the results obtained was not attributed to the drug administration method by subjecting the control animals to similar treatment. As such, the anticipated stress was evenly distributed between control and drug treatment groups.

## Conclusion

Celecoxib exhibited an attenuating effect on hyperlipidaemia and liver injury associated with sub-chronic ingestion of high fat (heated oil) in rats. Overall, findings from this study suggest that celecoxib, in addition to its established anti-inflammatory property, may be of therapeutic benefit in dyslipidaemia or related metabolic diseases and their attendant complications.

## Supporting information

S1 Data (XLSX)

## References

[1] Joo IW, Ryu JH, Oh HJ. The influence of Sam-Chil-Geun (Panax notoginseng) on the serum lipid levels and inflammations of rats with hyperlipidemia induced by poloxamer-407. Yonsei Med J. 2010; 51(4):504–10. doi: 10.3349/ymj.2010.51.4.504 20499414 PMC2880261

[2] Mc Namara K, Alzubaidi H, Jackson JK. Cardiovascular disease as a leading cause of death: how are pharmacists getting involved? Integr Pharm Res Pract. 2019;8:1–11. doi: 10.2147/IPRP.S133088 30788283 PMC6366352

[3] Kraakman MJ, Dragoljevic D, Kammoun HL, Murphy AJ. Is the risk of cardiovascular disease altered with anti-inflammatory therapies? Insights from rheumatoid arthritis. Clin Transl Immunol. 2016. 5(5);e84; doi: 10.1038/cti.2016.31 27350883 PMC4910124

[4] Stone NJ, Robinson J, Lichtenstein AH., Bairey Merz CN, Lloyd-Jones DM, Blum CB, et al. ACC/AHA guideline on the treatment of blood cholesterol to reduce atherosclerotic cardiovascular risk in adults: a report of the American College of Cardiology/American Heart Association Task Force on Practice Guidelines. J Am Coll Cardiol. 2013;63(25 Pt B):2889–934. doi: 10.1016/j.jacc.2013.11.002 24239923

[5] Perk J, De Backer G, Gohlke H, Graham I, Reiner Z, Verschuren M. European Association for Cardiovascular Prevention and Rehabilitation (EACPR), ESC Committee for Practice, et al. European guidelines on cardiovascular disease prevention in clinical practice (version 2012). The Fifth Joint Task Force of the European Society of Cardiology and Other Societies on Cardiovascular Disease Prevention in Clinical Practice (constituted by representatives of nine societies and by invited experts). Eur Heart J. 2012;13(13):635–701. 10.1093/eurheartj/ehs092 22555213

[6] WHO 2008. WHO. The World Health report. WHO; Geneva: 2008. Cardiovascular disease. www.who.int/cardiovascular.diseases/en/ [2019 September 9th].

[7] Mbikay M. Therapeutic potential of Moringa oleifera leaves in chronic hyperglycemia and dyslipidemia: a review. Front Pharmacol. 2012;3:24. doi: 10.3389/fphar.2012.00024 22403543 PMC3290775

[8] Nelson RH. Hyperlipidemia as a risk factor for cardiovascular disease. Prim Care. 2012;40(1):195–211. doi: 10.1016/j.pop.2012.11.003 23402469 PMC3572442

[9] Jou J, Choi SS, Diehl AM. Mechanisms of disease progression in nonalcoholic fatty liver disease. Semin Liver Dis. 2008;28:370–9. doi: 10.1055/s-0028-1091981 18956293

[10] Liao XH, Cao X, Liu J, Xie XH, Sun YH, Zhong BH. Prevalence and features of fatty liver detected by physical examination in Guangzhou. World J Gastroenterol. 2013;19(32):5334–9. doi: 10.3748/wjg.v19.i32.5334 23983438 PMC3752569

[11] Al Mamun A, Hashimoto M, Katakura M, Tanabe Y, Tsuchikura S, Hossain S, et al. Effect of dietary n-3 fatty acids supplementation on fatty acid metabolism in atorvastatin-administered SHR.Cg-Leprcp/NDmcr rats, a metabolic syndrome model. Biomed Pharmacother. 2017;85:372–9. doi: 10.1016/j.biopha.2016.11.038 27939244

[12] Rang HP, Ritter JM, Flower RJ, Henderson G. Rang & Dale’s Pharmacology E-Book: with STUDENT CONSULT Online Access. 2014. Elsevier Health Sciences.

[13] Kourounakis AP, Victoratos P, Peroulis N, Stefanou N, Yiangou M, Hadjipetrou L, et al. Experimental hyperlipidemia and the effects of NSAIDS. Exp Mol Pathol. 2002;73:135–8. doi: 10.1006/exmp.2002.2449 12231215

[14] Livshits A, Seidman DS. Role of Non-Steroidal Anti-Inflammatory Drugs in Gynecology. Pharmaceuticals. 2010;3:2082–9. doi: 10.3390/ph3072082 27713343 PMC4036657

[15] Ahmed S, Gul S, Zia-Ul-Haq M, Riaz M. Hypolipidemic effects of nimesulide and celecoxib in experimentally induced hypercholesterolemia in rabbits. Turk J Med Sci. 2015;45:277–83. doi: 10.3906/sag-1312-106 26084115

[16] Theodosis-Nobelos P., Kourti M., Gavalas A., & Rekka E. A. (2016). Amides of non-steroidal anti-inflammatory drugs with thiomorpholine can yield hypolipidemic agents with improved anti-inflammatory activity. Bioorg. Med. Chem. Lett, 26(3), 910–913. doi: 10.1016/j.bmcl.2015.12.063 26750253

[17] Theodosis-Nobelos P., Tziona P., Poptsis A., Athanasekou C., Kourounakis P. N., & Rekka E. A. (2017). Novel polyfunctional esters of ibuprofen and ketoprofen with hypolipidemic, lipoxygenase inhibitory and enhanced anti-inflammatory activity. Medicinal Chemistry Research, 26(2), 461–472.

[18] Dhawan V, Ganguly NK, Majumdar S, Chakavarti RN. Effect of indomethacin on serum lipids, lipoproteins, prostaglandins and the extent and severity of atherosclerosis in rhesus monkeys. Can J Cardiol. 1992; 8(3):306–12. 1576566

[19] Belton O, Fitzgerald DJ. Cyclooxygenase isoforms and atherosclerosis. Expert Rev Mol Med. 2003;5(9);1–18. doi: 10.1017/S1462399403005842 14987412

[20] FitzGerald GA, Patrono C. The coxibs, selective inhibitors of cyclooxygenase-2. N Engl J Med. 2001; 345(6): 433–42. doi: 10.1056/NEJM200108093450607 11496855

[21] Graham DJ, Campen D, Hui R, Spence M, Cheetham C, Levy G, et al. Risk of acute myocardial infarction and sudden cardiac death in patients treated with cyclo-oxygenase 2 selective and non-selective non-steroidal anti-inflammatory drugs: nested case-control study. Lancet. 2005;365(9458):475–81. doi: 10.1016/S0140-6736(05)17864-7 15705456

[22] Hudson M, Richard H, Pilote L. Differences in outcomes of patients with congestive heart failure prescribed celecoxib, rofecoxib, or non-steroidal anti-inflammatory drugs: population based study. *Bri Med J*. 2005;330(7504):1370. doi: 10.1136/bmj.330.7504.1370 15947399 PMC558290

[23] McGettigan P, Henry D. Cardiovascular risk and inhibition of cyclooxygenase: a systematic review of the observational studies of selective and nonselective inhibitors of cyclooxygenase 2. JAMA. 296(13), 1633–644. doi: 10.1001/jama.296.13.jrv60011 16968831

[24] Kristensen LE, Jakobsen AK, Askling J, Nilsson F, Jacobsson LT. Safety of etoricoxib, celecoxib, and nonselective nonsteroidal antiinflammatory drugs in ankylosing spondylitis and other spondyloarthritis patients: a Swedish national population‐based cohort study. Arthritis Care Res. 2015;67(8):1137–49. 10.1002/acr.22555 25623277

[25] Friend M, Vucenik I, Miller M. Platelet responsiveness to aspirin in patients with hyperlipidaemia. Br Med J. 2003;326:82–3. doi: 10.1136/bmj.326.7380.82 12521973 PMC139938

[26] Ekor M, Odewabi AO, Kale OE, Adesanoye OA, Bamidele TO. Celecoxib, a selective cyclooxygenase-2 inhibitor, lowers plasma cholesterol and attenuates hepatic lipid peroxidation during carbon-tetrachloride–associated hepatotoxicity in rats. Drug Chem Toxicol. 2013;36(1):1–8. doi: 10.3109/01480545.2011.642380 22168377

[27] Garber JC, Barbee RW, Bielitzki JT, Clayton L, Donovan J, Hendriksen C, et al. Guide for the care and use of laboratory animals. The National Academic Press, Washington DC 8: 220.

[28] Graham I, Shear C, Graeff PD, Boulton C, Catapano AL, Stough WG, et al. New strategies for the development of lipid-lowering therapies to reduce cardiovascular risk. Eur Heart J. 2018;4:119–27. doi: 10.1093/ehjcvp/pvx031 29194462

[29] Robinson JG, Huijgen R, Ray K, Persons J, Kastelein JJ, Pencina MJ. Determining when to add nonstatin therapy: a quantitative approach. J Am Coll Cardiol. 2016;68:2412–21. doi: 10.1016/j.jacc.2016.09.928 27908345

[30] Chen X, Xu S, Wei S, Deng Y, Li Y, Yang F, et al. Comparative Proteomic Study of Fatty Acid-treated Myoblasts Reveals Role of Cox-2 in Palmitate-induced Insulin Resistance. Sci Rep.2016; 6:21454. doi: 10.1038/srep21454 26899878 PMC4761885

[31] Yu J, Ip E, Dela Peña A, Hou JY, Sesha J, Pera N, et al. COX-2 induction in mice with experimental nutritional steatohepatitis: Role as pro-inflammatory mediator. Hepatology. 2006; 43:826–836. doi: 10.1002/hep.21108 16557554

[32] Chen J, Liu D, Bai Q, Song J, Guan J, Gao J, et al. Celecoxib attenuates liver steatosis and inflammation in non-alcoholic steatohepatitis induced by high-fat diet in rats. Mol Med Rep. 2011;4:811–816. doi: 10.3892/mmr.2011.501 21643627

[33] Adam SK, Das S, Soelaiman IN, Umar NA, Jaarin K. Consumption of repeatedly heated soy oil increases the serum parameters related to atherosclerosis in ovariectomized rats. Tohoku J Exp Med. 2008;215(3):219–26. doi: 10.1620/tjem.215.219 18648182

[34] de la Casa E, Perez-Gonzalez N, Sanchez-Bernal C, Llanillo M, Effects of dietary oil related to the toxic oil syndrome on the lipids of guinea pig liver microsomes. Lipids. 1995;30(6):575–9. doi: 10.1007/BF02537033 7651086

[35] Antwi-Baffour S, Kyeremeh R, Boateng SO, Annison L, Seidu MA. Haematological parameters and lipid profile abnormalities among patients with Type-2 diabetes mellitus in Ghana. Lipids Health Dis. 2018;17(1):1–9. doi: 10.1186/s12944-018-0926-y 30545361 PMC6293632

[36] Torregrosa JM, Ferrer-Marin F, Lozano ML, Moreno MJ, Martinez C, Anton AI, et al. Impaired leucocyte activation is underlining the lower thrombotic risk of essential thrombocythaemia patients with CALR mutations as compared with those with the JAK2 mutation. Bri J Haematol. 2016;172(5):813–815. doi: 10.1111/bjh.13539 26132594

[37] Longo M, Crosignani A, Podda M. Hyperlipidemia in Chronic Cholestatic Liver Disease. Curr Treat Options Gastroenterol. 2001;4(2):111–4. doi: 10.1007/s11938-001-0022-6 11469968

[38] Kalhan SC. Protein metabolism in pregnancy. Am J Clin Nutr 2000; 71, 5 Suppl: 1249S–55S. doi: 10.1093/ajcn/71.5.1249s 10799398

[39] Waring WS, Stephen AFL, Robinson ODG, Dow MA, Pettie JM. Serum urea concentration and the risk of hepatotoxicity after paracetamol overdose. QJM. 2008;101(5):359–63. doi: 10.1093/qjmed/hcn023 18334496

[40] Skalen K, Gustafsson M, Rydberg EK. Subendothelial retention of atherogenic lipoproteins in early atherosclerosis. Nature. 2002;417:750–4. doi: 10.1038/nature00804 12066187

[41] Reilly MP, Pratico D, Delanty N, DiMinno G, Tremoli E, Rader. Increased formation of distinct F2 isoprostanes in hypercholesterolemia. Circulation. 1998; 98(25):2822–8. doi: 10.1161/01.cir.98.25.2822 9860782

[42] Ross R. The pathogenesis of atherosclerosis: a perspective for the 1990s. Nature. 1993;362(6423):801–9. doi: 10.1038/362801a0 8479518

[43] Evereklioglu C, Er H, Türköz Y, Cekmen M. Serum levels of TNF-alpha, sIL-2R, IL-6, and IL-8 are increased and associated with elevated lipid peroxidation in patients with Behçet’s disease. Mediators Inflamm. 2002;11(2):87–93. doi: 10.1080/09629350220131935 12061429 PMC1781647

[44] Sellers RS, Mortan D, Michael B, Roome N, Johnson JK, Yano BL. Society of Toxicologic Pathology position paper: organ weight recommendations for toxicology studies. Toxicol Pathol. 2007;35(5):751–5. doi: 10.1080/01926230701595300 17849358

[45] Gaw A, Murphy M, Srivastava R, Cowan RA, O’Reilly DSJ. Clinical Biochemistry E-Book: An Illustrated Colour Text: Elsevier Health Sciences. 2013.

[46] Contreras-Zentella ML, Hernández-Muñoz R. Is liver enzyme release really associated with cell necrosis induced by oxidant stress?. Oxid. Med. Cell. Longev. 2016; 2016: 1–12. doi: 10.1155/2016/3529149 26798419 PMC4699024

